# Supplementary material for: Association between Environmental Cadmium Exposure and Osteoporosis Risk in Postmenopausal Women: A Systematic Review and Meta-Analysis
Source: Int J Environ Res Public Health. 2022 Dec 28;20(1):485. doi: 10.3390/ijerph20010485 (PMC9820024; doi:10.3390/ijerph20010485)
Supplement: Supplementary file 1 [file ijerph-20-00485-s001.zip › ijerph-2059517-supplementary.pdf]

Supplementary Materials

Association between environmental cadmium exposure and osteoporosis risk in postmenopausal women: a systematic review and meta-analysis

Carlos Tadashi Kunioka[1,2], Maria Conceição Manso[1,3,4], Márcia Carvalho[1,3,5,6,*]

[1] FP-I3ID, FP-BHS, University Fernando Pessoa, 4249-004 Porto, Portugal

[2] Western Paraná State University (UNIOESTE), 85819-110 Cascavel, Paraná, Brazil

[3] Faculty of Health Sciences, University Fernando Pessoa, 4200-150 Porto, Portugal

[4] LAQV, REQUIMTE, University of Porto, 4050-313 Porto, Portugal

[5] Associate Laboratory i4HB-Institute for Health and Bioeconomy, Faculty of Pharmacy, University of Porto, 4050-313 Porto, Portugal

[6] UCIBIO-REQUIMTE, Laboratory of Toxicology, Department of Biological Sciences, Faculty of Pharmacy, University of Porto, 4050-313 Porto, Portugal

* Correspondence: mcarv@ufp.edu.pt

Address:
Faculty of Health Sciences
University Fernando Pessoa
Rua Carlos da Maia, 296
4200-150 Porto, Portugal
Tel.: +351 225071300

Table S1. PubMed search strategy.

Database	Search strategy	Search results
PubMed	(("cadmium"[MeSH Terms] OR "cadmium"[All Fields]) AND ("womans"[All Fields] OR "women"[MeSH Terms] OR "women"[All Fields] OR "woman"[All Fields] OR "women s"[All Fields] OR "womens"[All Fields] OR ("womans"[All Fields] OR "women"[MeSH Terms] OR "women"[All Fields] OR "woman"[All Fields] OR "women s"[All Fields] OR "womens"[All Fields]) OR ("femal"[All Fields] OR "female"[MeSH Terms] OR "female"[All Fields] OR "females"[All Fields] OR "female s"[All Fields] OR "females"[All Fields])) AND ("bone density"[MeSH Terms] OR ("bone"[All Fields] AND "density"[All Fields]) OR "bone density"[All Fields]) AND ("environmental exposure"[All Fields] OR ("environ"[All Fields] OR "environment"[MeSH Terms] OR "environment"[All Fields] OR "environments"[All Fields] OR "environment s"[All Fields] OR "environs"[All Fields])) AND 2008/01/01:2021/12/31[Date - Publication]) NOT (("child"[MeSH Terms] OR "child"[All Fields] OR "children"[All Fields] OR "child s"[All Fields] OR "children s"[All Fields] OR "childrens"[All Fields] OR "childs"[All Fields]) AND ("child"[MeSH Terms] OR "child"[All Fields] OR "children"[All Fields] OR "child s"[All Fields] OR "children s"[All Fields] OR "childrens"[All Fields] OR "childs"[All Fields]) AND ("animals"[MeSH Terms:noexp] OR "animal"[All Fields]))	44

Table S2. Individual quality assessment of studies included in the systematic review using the Joanna Briggs Institute (JBI) critical appraisal checklist for cross-sectional studies.

Study	Q1	Q2	Q3	Q4	Q5	Q6	Q7	Q8	%	Risk
Gallagher *et al.* 2008	✓	✓	✓	✓	✓	✓	✓	✓	100	Low
Engstrom *et al.* 2009	✓	✓	✓	✓	X	X	✓	✓	75	Low
Horiguchi *et al.* 2010	✓	✓	✓	✓	X	X	✓	✓	75	Low
Suwazono *et al.* 2010	✓	✓	✓	✓	✓	✓	✓	✓	100	Low
Chen *et al*. 2011	✓	✓	✓	✓	✓	✓	✓	✓	100	Low
Engstrom *et al.* 2011	✓	✓	✓	✓	✓	✓	✓	✓	100	Low
Kim *et al.* 2014	✓	✓	✓	✓	✓	✓	✓	✓	100	Low
Callan *et al.* 2015	✓	✓	✓	✓	✓	✓	✓	✓	100	Low
Lv *et al.* 2017	✓	✓	✓	✓	✓	✓	✓	✓	100	Low
La-Up *et al.* 2021	✓	✓	✓	✓	✓	✓	✓	✓	100	Low

Q1. Were the criteria for inclusion in the sample clearly defined?; Q2. Were the study subjects and the setting described in detail?; Q3. Was the exposure measured in a valid and reliable way?; Q4. Were objective, standard criteria used for measurement of the condition?; Q5. Were confounding factors identified?; Q6. Were strategies to deal with confounding factors stated?; Q7. Were the outcomes measured in a valid and reliable way?; Q8. Was appropriate statistical analysis used?

'✓' indicates yes, and '✕' indicates no

Table S3. Egger´s test of studies investigating the association between UCd levels and risk of osteoporosis in the high-level Cd exposure group.

Egger's Regression-Based Test[a]

Parameter	Coefficient	Std. Error	t	Sig. (2-tailed)	95% Confidence Interval	
					Lower	Upper
SE[b]	-4.430	16.3412	-0.271	0.831	-212.065	203.204
Bias	0.001	0.0015	0.692	0.166	-0.019	0.020

[a] Random-effects meta-regression
[b] Standard error of effect size
